# Naturalistic driving measures of route selection associate with resting state networks in older adults

**DOI:** 10.1038/s41598-022-09919-x

**Published:** 2022-04-20

**Authors:** Julie K. Wisch, Catherine M. Roe, Ganesh M. Babulal, Nicholas Metcalf, Ann M. Johnson, Samantha Murphy, Jamie Hicks, Jason M. Doherty, John C. Morris, Beau M. Ances

**Affiliations:** 1 Department of Neurology, Washington University in Saint Louis School of Medicine, 660 South Euclid Avenue, Campus Box 8111, St. Louis, MO 63110 USA; 2 Department of Radiology, Washington University in St. Louis, St. Louis, MO 63110 USA; 3 Knight Alzheimer Disease Research Center, Washington University School of Medicine, St Louis, MO 63110 USA; 4 Department of Clinical Research and Leadership, The George Washington University School of Medicine and Health Sciences, Washington, DC USA; 5 Department of Psychology, Faculty of Humanities, University of Johannesburg, Johannesburg, South Africa; 6 Center for Clinical Studies, Washington University in St. Louis, St. Louis, MO 63110 USA

**Keywords:** Neurodegeneration, Cognitive ageing

## Abstract

Our objective was to identify functional brain changes that associate with driving behaviors in older adults. Within a cohort of 64 cognitively normal adults (age 60+), we compared naturalistic driving behavior with resting state functional connectivity using machine learning. Functional networks associated with the ability to interpret and respond to external sensory stimuli and the ability to multi-task were associated with measures of route selection. Maintenance of these networks may be important for continued preservation of driving abilities.

## Introduction

Movement ecology is a broad paradigm seeking to analyze how, why, and where organisms move^1^. As individuals age, functional movement changes can be observed^2,3^; these changes can be attributed to structural and functional changes in the brain^3^. Struggles with navigation in older adults are well documented^4,5^, and changes in movement as demonstrated by differences in how individuals move around an assisted living facility have also been found^2^. Here we collected naturalistic driving data over a multi-year period in a cohort of cognitively normal older adults, giving us a very large volume of observational movement behavior data. We also collected resting state (rsfc) functional MRI (fMRI) on these individuals at a single time point, so that we could begin to evaluate the relationships between brain function and movement patterns in the aging adult.

The literature linking human brain function to driving-based movement primarily relies on simulation studies^6–9^. A diverse set of functional brain networks, including attention, motor control, decision making, and the sensory networks have been shown to be activated. However, only one study has evaluated rsfc as a function of driving skill. The study compared taxi drivers (skilled drivers) and non-drivers, finding that taxi drivers possessed stronger connections integrating higher order control networks with sensory networks, and weaker connections between sensory networks^10^.

Notably, none of these aforementioned studies consider aging-related changes that may occur in drivers. Drivers do experience changes as they age; older drivers have an increased risk of crashes that may be due to age-related changes in sensorimotor processing in combination with difficulties in spatial navigation^4,11^. The average older adult ceases driving about 7 years prior to the end of their lives^12,13^. For individuals who stop driving, many will develop depression, lower social participation and physical activity, and increased risk of transition to institutional care^14^. Interventions empowering older adults to continue driving could have important societal benefits.

We were interested in functional brain changes that associate with driving behaviors in older adults, so we examined rsfc in 64 cognitively normal older adults and looked for associations with naturalistic driving data. Note that the primary question associated with navigation while driving is “what does the movement path of directed travel look like?”^2^ For this reason, we included two new measures: straightness and actual-optimal distance ratio. Straightness is a metric that appears regularly in movement ecology literature^2,15,16^, and has been applied specifically to humans^2^, although never before in a driving context. We developed the actual-optimal distance ratio as a hedge against environmental constraints, and this metric is discussed in further detail in the results and conclusion portion of the paper. We applied machine learning to identify rsfc networks that associate with many driving behaviors, including the two navigational measures highlighted here. This study is an important addition to the field’s understanding of the functional changes that associate with aging, as we are able to connect naturalistic movement data with neuroimaging data.

## Methods

### Participants

Cognitively normal older adults (n = 64, age ≥ 60) enrolled in a driving study affiliated with the Knight Alzheimer Disease Research Center (ADRC) at Washington University in St. Louis were included in this analysis. Methods of recruitment have been previously described^17,18^. All participants undergo regular cognitive screening, and all participants also completed vision screening. This study was approved by the WUSTL Institutional Review Board (IRB # 202010214 and 201706043), and each participant provided signed informed consent. All methods were carried out in accordance with relevant guidelines and regulations.

### Imaging

We obtained both structural and functional MRI on a 3 T Siemens biograph scanner (Erlangen, Germany). We used a seed-based approach for rsfc. After calculating the mean time series for each functional region of interest (ROI), we calculated the pairwise correlations between each ROI. Then we sorted these seeds into 13 pre-defined networks to develop a 13 × 13 matrix of inter- and intra-network connections^19^. All imaging data was collected within 2.5 years of driving study enrollment. Scans were collected once per participant with the timeframe of collection shown in Supplemental Fig. 1. Extended details regarding image acquisition are available in the Supplemental Materials.

### Driving

Naturalistic driving was captured using the Driving Real-World In-Vehicle Evaluation System (DRIVES)^17,18^. This data was obtained through the use of a GPS data logger (G2 Tracking Device, Azuga Inc, San Jose, CA), installed in the onboard diagnostics-II port of a vehicle. Date, time, speed, latitude and longitude coordinates at 30-s epochs were collected whenever a vehicle drove. In addition to the breadcrumb data, trip summary values including total distance travelled, counts of hard braking incidents, hard accelerations, and duration exceeding the speeding limit for each trip were collected. All participants supplied more than 1 year of driving data ($\mu_{{\text{duration of enrollment}}}$ = 3.01 years, $\sigma_{{\text{duration of enrollment}}}$ = 1.06 years). Detailed information on participant enrollment duration and time between rsfc collection and driving study participation is available in Supplemental Materials.

### Statistics

**Figure 1:**
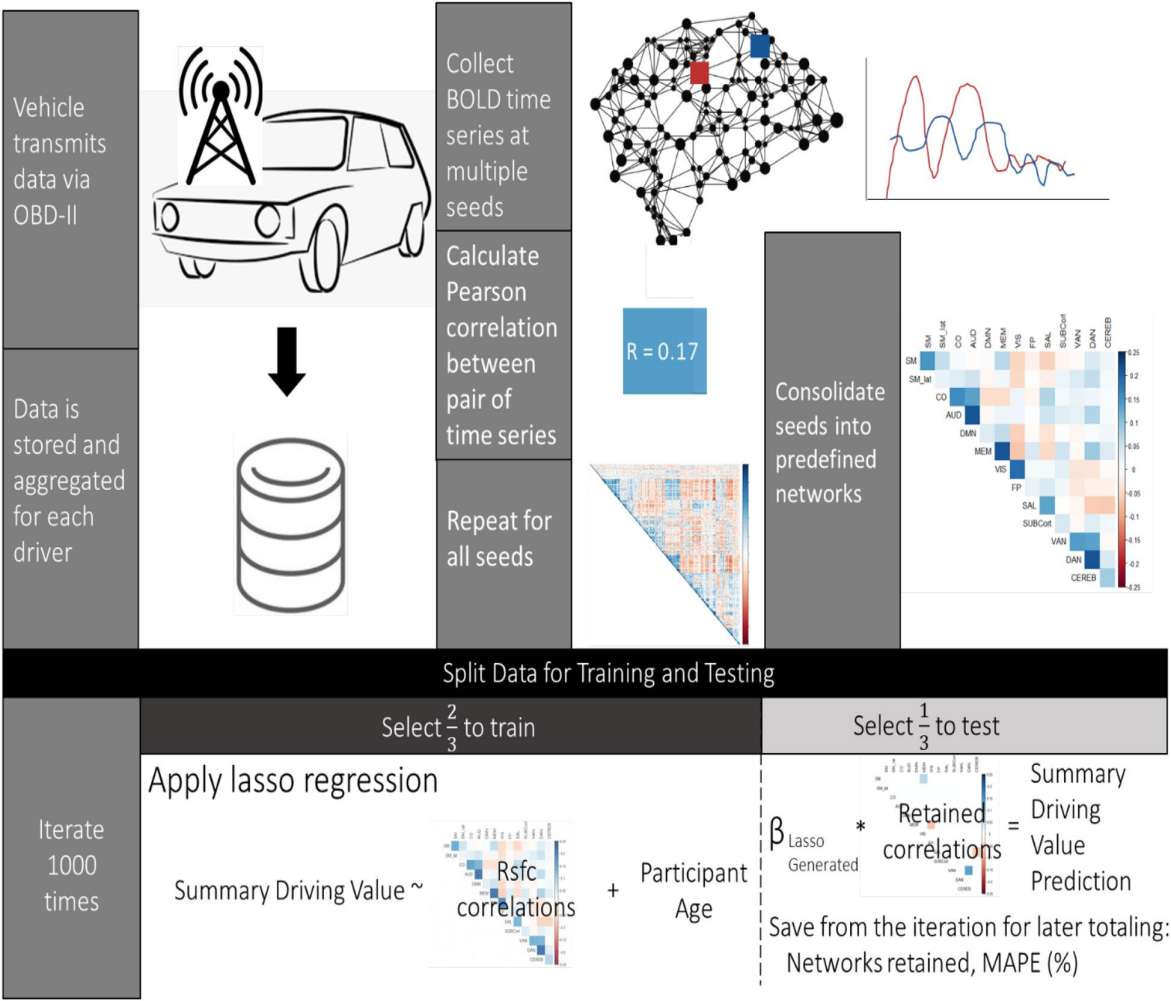
Vehicle data was collected via chip transmission (OBD-II), and single summary values were created for each measurement on a driver-by-driver basis. Resting state functional connectivity (rsfc) functional magnetic resonance imaging (fMRI) was collected with time series data and converted to a matrix of correlations, consistent with previously published methodology. We then used a 1000 bootstrap procedure to identify rsfc networks that predicted driving performance variables. For each iteration, we trained the model on two-thirds of the dataset and evaluated the model performance on the remaining one third. From each iteration we kept the networks that were retained by the lasso algorithm and the mean average percent error (MAPE) of the proposed model. At the conclusion of the 1000 iterations, we counted the total number of times each network was retained and calculated the average MAPE.

Driving parameters (complete list available in Table 1) were compressed into single values for each driver over their entire recorded driving history. We performed a 1000 bootstrap lasso regression with tenfold cross-validation for lambda selection, similar to a previously described approach^20^. We applied the R package glmnet for implementation^21^. For each bootstrap iteration, we trained on 2/3 of the data and tested on the remaining 1/3. We took each of the driving parameters in turn as the response variable, and included each rsfc correlation value and driver age as features in the model. We looked for models with mean absolute percent error (MAPE) of less than 10% indicating good forecasting power. In order to identify the features that contributed the most to generating good predictions, we counted the number of times each feature was retained by lasso regression (see Fig. 1).

**Table 1:** Participant Demographic Data.

	Participants (N = 64)
**Age at MRI (years)**	
Mean (SD)	71.3 (5.06)
Median [Min, Max]	70.3 [60.0, 84.7]
**Sex**	
Male	32 (50%)
Female	32 (50%)
**Education (years)**	
Mean (SD)	16.6 (2.19)
Median [Min, Max]	16.0 [12.0, 20.0]
**Time from start of driving enrollment to MRI (years)**	
Mean (SD)	0.997 (1.01)
Median [Min, Max]	1.03 [− 2.00, 2.49]
**Time from end of driving enrollment to MRI (years)**	
Mean (SD)	4.01 (1.03)
Median [Min, Max]	3.91 [1.69, 6.55]
**Radius of gyration (mi)**	
Mean (SD)	257.4 (251.4)
Median [Min, Max]	177.6 [18.54, 1146]
**Median distance travelled (mi)**	
Mean (SD)	4.12 (2.40)
Median [Min, Max]	3.29 [0.684, 14.3]
**Median route straightness**	
Mean (SD)	0.718 (0.0535)
Median [Min, Max]	0.725 [0.589, 0.838]
**Median actual-optimal distance ratio**	
Mean (SD)	1.00 (0.0266)
Median [Min, Max]	0.999 [0.939, 1.10]
**Median actual-optimal time ratio**	
Mean (SD)	1.32 (0.274)
Median [Min, Max]	1.25 [0.940, 2.15]
**Number of trips per year**	
Mean (SD)	619 (360)
Median [Min, Max]	565 [120, 1950]
**Mean number of hard braking events per trip**	
Mean (SD)	0.110 (0.0882)
Median [Min, Max]	0.0931 [4.80e−03, 0.509]
**Mean number of hard acceleration events per trip**	
Mean (SD)	0.0423 (0.101)
Median [Min, Max]	0.0127 [0, 0.746]
**Mean number of overspeeding events per trip**	
Mean (SD)	0.394 (0.422)
Median [Min, Max]	0.288 [0, 1.94]
**Mean percentage of trip time spent overspeeding**	
Mean (SD)	0.0122 (0.0168)
Median [Min, Max]	6.76e-03 [0, 0.0845]
**Mean number of unique destinations per year**	
Mean (SD)	201 (103)
Median [Min, Max]	187 [50.5, 520]
**Ratio of unique destinations to total destinations**	
Mean (SD)	0.341 (0.0811)
Median [Min, Max]	0.335 [0.208, 0.530]

## Results/discussion/conclusion

After evaluating a variety of driving behavioral metrics, we found strong predictability for two measures (straightness and actual-optimal distance ratio) with regards to rsfc networks. Complete results are available in Supplemental Table 1. Not all driving parameters considered were related to navigation; however, both parameters that demonstrated strong associations with rsfc were navigation-related parameters. We observed positive correlations between within-network ventral attention network (VAN × VAN) and salience–dorsal attention network (SAL × DAN) and straightness, as well as a positive correlation between frontoparietal–subcortical network (FP × SubCort) and the actual-optimal distance ratio in this sex-balanced cohort of cognitively normal older adults.

**Figure 2:**
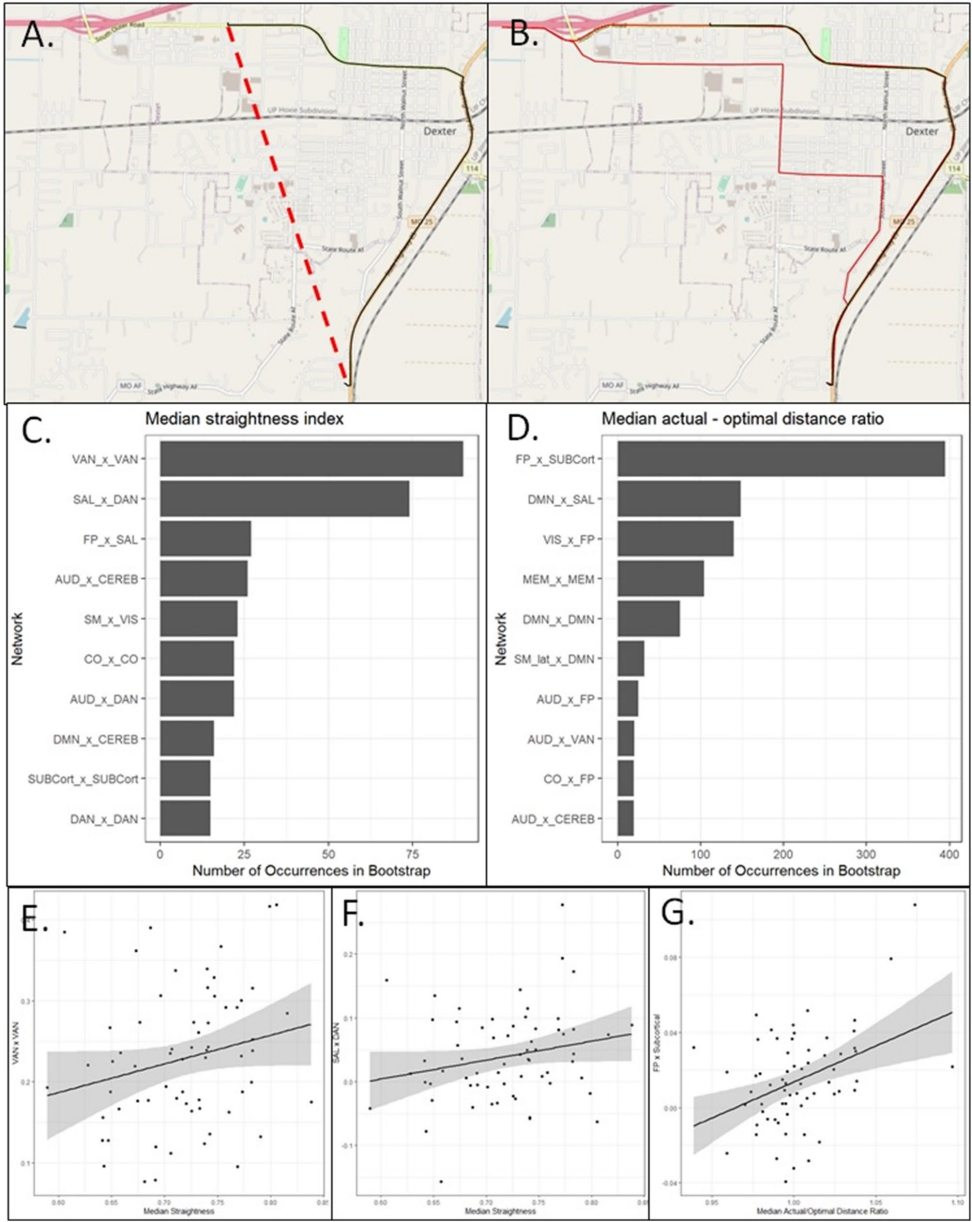
(**A**) Route straightness was calculated by taking the Haversine distance (shown in red) divided by the actual route (shown in black) distance. This figure was generated using Open Street Maps data, available under Creative Commons License 2.0 (https://www.openstreetmap.org/copyright; Figure generated 2022 Jan 18) and the R packages osrm and mapsf. (**B**) The actual–optimal distance ratio was calculated by dividing the length of the actual route (shown in black) divided by the optimal route (shown in red). The actual route driven was estimated using the coordinates from each 30 s epoch breadcrumbs from the ODB-II chip. The optimal route was generated via Open Street Maps (https://www.openstreetmap.org/copyright; Figure generated 2022 Jan 18). For this example, the actual route driven was six miles, while the optimal proposed route was four miles. The actual–optimal distance ratio for this particular route was 1.5. (**C**) The networks most frequently utilized for prediction of the median straightness index were the intranetwork ventral attention network connection (VAN × VAN) and the salience–dorsal attention network connection (SAL × DAN). (**D**) The network most frequently utilized for prediction of the median actual–optimal distance ratio was the frontoparietal–subcortical (FP × SubCort) network connection. (**E**) There was a positive correlation between the VAN × VAN connection and median route straightness. (**F**) There was a positive correlation between the SAL × DAN connection and median route straightness. (**G**) There was a positive correlation between the FP × SubCort connection and the median actual–optimal distance ratio.

In animal movement literature, maximum route straightness (straightness = 1) is considered the most efficient orientation posture; however, even in the natural world, animals frequently are not able to follow perfectly direct routes due to any number of obstacles posed by the physical environment or potential predators^16^. By extension, drivers are constrained by transportation infrastructure and it is thus unlikely that they are able to select perfectly straight routes. We considered higher straightness to represent more optimal route finding, even given environmental constraints, consistent with prior applications in human movement ecology^2^. It is possible that low straightness values would indicate drivers who are getting lost more frequently and exhibiting avoidant driving behaviors that lead them to take longer and/or more circuitous routes, such as the route shown in Fig. 2B. In Fig. 2B we see a driver who took a much longer and circuitous route (shown in black) in order to avoid a highway (the optimal route, shown in red).

Median route straightness (Fig. 2A) could be predicted with 6.4% MAPE. A strong VAN × VAN connection was associated with greater route straightness (Fig. 2C,E). The VAN is responsible for bottom up attentional processing^22,23^, specifically, for exercising control upon receipt of various extrasensory stimuli. The SAL × DAN inter-network connection also positively correlated with route straightness (Fig. 2D,F). The SAL × DAN network has been shown to associate with motor inhibition^24^. Taken together, we see that the two most important networks used for predicting route straightness were associated with the ability to respond to a complex external environment, including the ability to alter pre-planned motor activities.

Despite the utility described above, we acknowledge that utilizing straightness as our primary route selection metric could be heavily influenced by the availability of roads. Therefore, we used OpenStreetMap to identify the theoretically optimal route, which optimizes routing by predicted travel duration, meaning that it finds the route that should be quickest rather than shortest distance^25^. Our working assumption was that drivers who take longer routes than the optimal route were either getting lost, choosing to drive in primarily familiar areas even if they were not the most direct path from point A to point B, or intentionally avoided challenging driving scenarios like high speed environments (again, see Fig. 2B) or complex intersections. We were able to predict the median actual–optimal distance ratio with 2.0% MAPE.

We observed a positive correlation between our primary resting state network, this time FP × SubCort, and the driving metric in question (Fig. 2G). Lower FP × SubCort values indicate a greater proficiency at multi-tasking^26^. Drivers who exhibited greater multi-tasking ability based on their rsfc values had lower actual-optimal distance ratios, suggesting that the ability to multi-task is important to the ability to consistently drive more direct routes. Notably, FP × SubCort changes occur with cognitive training^26^, meaning that FP × SubCort targeted interventions could potentially lead to longer retention of access to personal vehicles for older adults.

We expected straightness and the actual-optimal distance ratio to contain similar information; however, they were relatively weakly correlated (R = 0.22). These results suggest that different information is conveyed by the straightness index and the actual-optimal distance ratio, and highlights a major limitation of this work. Although both metrics seem to relate to wayfinding in a naturalistic driving setting, we are not able to determine if either (or both) are reflective of intentional route selections (e.g. drivers choosing to avoid complex driving scenarios) or errors in wayfinding. If these factors could be isolated, we could potentially derive even greater insight into the neurological underpinnings of different features of driving with aging. Future work that investigates individual routes rather than aggregated median values is necessary to enhance our understanding of these differences.

We observed that for older adults, retention of strong networks associated with the ability to interpret and respond to external sensory stimuli and the ability to multi-task played important roles in the route selection process. This general pattern aligns with the observed result of stronger connections between control and sensory networks for drivers^10^. Defining these relationships is the first step to the development of targeted interventions like cognitive training. This information could also inform the work of driving rehabilitation specialists; more deeply understanding specific deficits that older drivers may struggle with could enhance transfer learning efforts to driving in their own vehicles^27^. Longer retention of access to personal vehicles could increase the number of older adults that could age-in-place, as well as maintain the sense of autonomy that comes with the ability to independently navigate. This is an emerging field of literature and more work is required before concrete steps can be recommended.

## Supplementary Information

Supplementary Information.
