# Supplementary material for: Naturalistic driving measures of route selection associate with resting state networks in older adults
Source: Sci Rep. 2022 Apr 20;12:6486. doi: 10.1038/s41598-022-09919-x (PMC9021301; doi:10.1038/s41598-022-09919-x)
Supplement: Supplementary file 1 — Supplementary Information. [file 41598_2022_9919_MOESM1_ESM.pdf]

Supplemental Materials

Supplemental Figure 1. Enrollment in driving study is shown for individual participant. Resting state functional connectivity (rsfc) was collected at year 0. All individuals contributed at least one year of driving data and exactly one rsfc scan. Individuals with the longest horizontal lines contributed driving data for the longest interval.

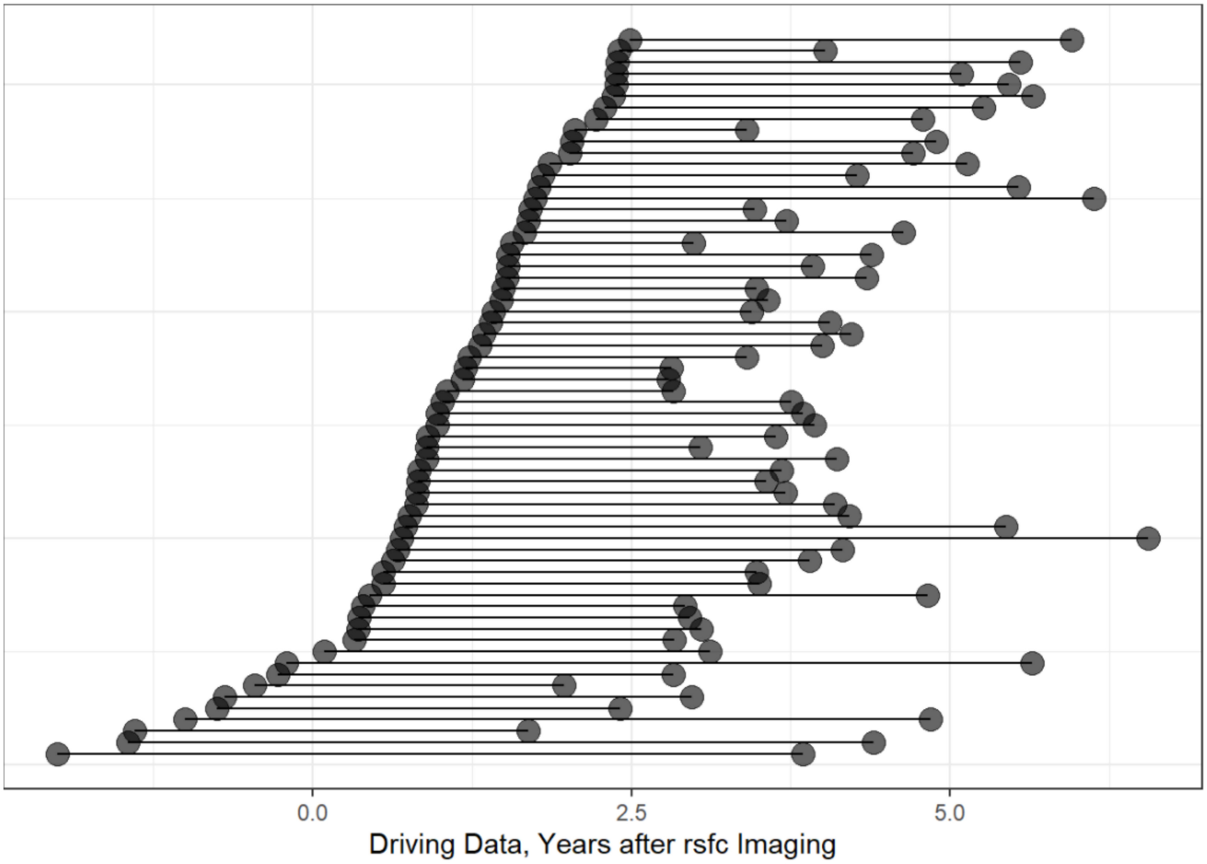

Supplemental Table 1. We attempted to forecast all 15 driving parameters displayed in Table 1 using only rs-fc composite scores as predictors. Only two parameters, as discussed in the main body of the manuscript, were forecast with less than 10% mean average percent error (MAPE). Here we present error metrics for all 15 parameters, showing Mean Average Error (MAE), Root Mean Squared Error (RMSE) and MAPE. The other parameter that could be predicted with moderate success was the median actual-optimal time ratio. This value was moderately correlated with the median actual-optimal distance ratio ($R_{Spearman}$ = 0.245, p = 0.051), but not correlated with median route straightness ($R_{Spearman}$ = -0.065, p = 0.610).

	**MAE**	**RMSE**	**MAPE**
**Radius of Gyration**	302.46 mi	414.01 mi	168.22%
**Median Distance Travelled (mi)**	1.85 mi	2.48 mi	58.27%
**Median Route Straightness**	0.045	0.055	6.36%
**Median Actual-Optimal Distance Ratio**	0.020	0.027	2.00%
**Median Actual-Optimal Time Ratio**	0.22	0.27	16.08%
**Number of Trips per Year**	268.34	373.04	60.16%
**Mean number of hard braking events per trip**	0.066	0.092	120.00%
**Mean number of hard acceleration events per trip**	0.048	0.089	>200%
**Mean number of overspeeding events per trip**	0.34	0.44	>200%
**Mean percentage of trip time spent overspeeding**	0.012	0.017	>200%
**Mean number of unique destinations per year**	82.69	106.44	53.02%
**Ratio of unique destinations to total destinations**	0.071	0.085	22.46%

Supplemental Text

MRI Imaging – Structural

We collected MRI using 3T Siemens scanners (Erlangen, Germany) with a standard 12-channel head coil. Structural images were acquired using the Alzheimer's Disease Neuroimaging Initiative (ADNI) protocol. DICOM files were transferred from the scanners to a local server[1]. We applied quality assurance of de-identified data. The study coordinator validated the sequence parameters during scanning, and we performed recapture if the session was corrupted by motion. All MRI scans are examined by a board-certified neuroradiologist at Washington University in St. Louis (WUSTL) for abnormalities.

We processed MRI images with FreeSurfer as detailed previously[2]. We applied motion correction and intensity normalization prior to segmenting both grey- and white matter structures from a T1 weighted image. We registered images to a spherical atlas and parcellated the cerebral cortex using the Desikan atlas[3]. We also applied partial volume correction in order to correct for head size[4].

MRI Imaging – Resting State Functional Connectivity

We collected resting state functional connectivity (rsfc) scans during the same session as the aforementioned T1 weighted structural MRI scan. Participants were instructed to lie still with their eyes open, and avoid falling asleep. We collected 36 contiguous slices in the sagittal direction[5] and processed data consistent with previously published work[5,6].

We corrected for slice intensity differences and corrected for intensity inhomogeneity using FSL FAST. We corrected for echoplanar imaging (EPI) distortion using a mean field map. We registered the EPI mean image to the atlas via MP-RAGE. We compensated for head motion, distortion correction and atlas transformation sequentially in order to produce a volumetric time series.

We classified excessive head motion as either a DVARS $> 0.9\%$ root mean square of the frame-to-frame signal change from the entire brain or frame displacement > 0.3 mm. We censored the frames if either of the criterion were exceeded. We applied band pass filtering to retain frequencies between 0.005 Hz and 0.1 Hz. We applied global signal regression and then used FNIRT to non-linearly warped the preprocessed time series data to the Montreal Neurological Institute 152 (3 mm^3 voxels) space atlas.

1. Marcus, D. S., Archie, K. A., Olsen, T. R. & Ramaratnam, M. The open-source neuroimaging research enterprise. *Journal of Digital Imaging* **20**, 130–138 (2007).
2. Fischl, B. FreeSurfer. *NeuroImage* vol. 62 774–781 (2012).
3. Desikan, R. S. *et al.* An automated labeling system for subdividing the human cerebral cortex on MRI scans into gyral based regions of interest. *Neuroimage* **31**, 968–980 (2006).
4. Buckner, R. L. *et al.* A unified approach for morphometric and functional data analysis in young, old, and demented adults using automated atlas-based head size normalization: Reliability and validation against manual measurement of total intracranial volume. *Neuroimage* **23**, 724–738 (2004).
5. Thomas, J. B. *et al.* Functional connectivity in autosomal dominant and late-onset Alzheimer disease. *JAMA Neurology* **71**, 1111–1122 (2014).
6. Brier, M. R. *et al.* Loss of intranetwork and internetwork resting state functional connections with Alzheimer's disease progression. *Journal of Neuroscience* **32**, 8890–8899 (2012).
